# Main branches pulmonary artery stenting in congenital heart diseases: a case series

**DOI:** 10.3389/fcvm.2026.1732330

**Published:** 2026-03-04

**Authors:** Radityo Prakoso, Yovi Kurniawati, Sisca Natalia Siagian, Aditya Agita Sembiring, Damba Dwisepto Aulia Sakti, Brian Mendel, Olfi Lelya, Oktavia Lilyasari

**Affiliations:** 1 Division of Pediatric Cardiology and Congenital Heart Disease, Department of Cardiology and Vascular Medicine, National Cardiovascular Center Harapan Kita, Universitas Indonesia, Jakarta, Indonesia; 2 Department of Cardiology and Vascular Medicine, National Cardiovascular Center Harapan Kita, Universitas Indonesia, Jakarta, Indonesia

**Keywords:** catheter intervention, congenital heart disease, Fontan circulation, pulmonary artery stenosis, stent

## Abstract

**Background:**

Branch pulmonary artery (PA) stenosis is a common complication in congenital heart disease (CHD) that can result in unequal pulmonary perfusion, cyanosis, and increased ventricular workload. Although surgical repair remains an option, pulmonary artery stenting has emerged as a less invasive and effective alternative for restoring vessel patency.

**Methods:**

This single-center retrospective study included 18 pediatric patients (median age 5.4 years, range 3 months–17 years) who underwent PA stenting for branch stenosis associated with complex CHD. Patient demographics, procedural characteristics, complications, and follow-up outcomes were analyzed.

**Results:**

A total of 19 stents were successfully implanted; 10 in the left PA, 7 in the right PA, and one case of bilateral stenting. The mean pre-procedural oxygen saturation improved from 78.1% ± 12.5 to 91.6% ± 7.3 during follow-up. Procedural success was achieved in all cases. Five complications were recorded, including one case of stent dislodgement requiring surgical retrieval and one case of inadequate stent expansion due to over-compliant pulmonary arteries. Three deaths occurred, all attributed to underlying clinical deterioration rather than the procedure itself. No instances of vascular rupture or pericardial tamponade were observed. Most surviving patients demonstrated sustained improvement in oxygenation and progressed to definitive surgical repair or Fontan completion.

**Conclusions:**

Pulmonary artery stenting is a safe and effective intervention for managing branch PA stenosis in complex pediatric CHD, providing significant hemodynamic and clinical benefits with an acceptable complication profile. Careful pre-procedural imaging, appropriate stent selection, and meticulous deployment technique are essential to prevent complications such as dislodgement or incomplete expansion, ensuring durable long-term outcomes.

## 1 Introduction

Branch pulmonary artery (PA) stenosis is a common and clinically significant complication in pediatrics with congenital heart disease (CHD), either as part of the native malformation or secondary to surgical or catheter-based palliation (1, 2). The resulting obstruction leads to unequal pulmonary perfusion, persistent cyanosis, and increased ventricular workload, which can ultimately compromise ventricular function and the success of subsequent surgical stages such as the Fontan procedure or complete repair (3, 4). While surgical reconstruction has traditionally been the mainstay of treatment, it is often technically demanding, particularly in reoperative settings, due to adhesions, limited access to distal branches, and a high risk of restenosis. These limitations have prompted the development of less invasive alternatives to restore pulmonary flow and improve clinical outcomes (1, 4).

Over the past two decades, pulmonary artery stenting has emerged as a safe and effective alternative to surgical reintervention for branch PA stenosis (3). By providing sustained vessel patency and preventing elastic recoil, PA stenting offers durable hemodynamic improvement and has become an essential component of staged CHD management. It is frequently employed as both a definitive and preparatory intervention following Blalock–Taussig (BT) shunt, PDA stent, right ventricular outflow tract (RVOT) stenting, bidirectional cavopulmonary shunt (BCPS), or Fontan patients where optimizing pulmonary flow distribution is crucial for subsequent surgery (5–7). Nevertheless, the procedure remains technically demanding due to small vessel size, complex anatomy, and prior surgical modifications, and carries risks including stent migration, thrombosis, and reperfusion injury. The reported incidence of serious adverse events (AEs) associated with pulmonary artery stenting is relatively low, occurring in less than 5% of cases (8–10), whereas balloon angioplasty demonstrates a broader complication rate ranging from 4% to 18% (11, 12). Given the scarcity of large multicenter data and the variability in practice, single-center experiences remain vital to improving procedural strategies and outcomes. Therefore, this study aims to report our institutional experience with pulmonary artery stenting in complex pediatric CHD, focusing on procedural success, complications, and long-term clinical impact.

## 2 Methods

This study is a retrospective case series in which patients were identified from the institutional catheterization laboratory database, including all consecutive pediatric patients who required left or right pulmonary artery (LPA/RPA) stenting during the study period from March 2021 to September 2025. Clinical, procedural, and outcome data were extracted from medical records, catheterization reports, and procedural documentation, with follow-up from the index procedure until the last available clinical visit within the study period. The primary outcome was patient survival, while secondary outcomes included procedure-related and post-procedural complications. Formal statistical analysis was not performed due to the very small number of subjects, and results are therefore presented descriptively. Ethical committee approval was not obtained because of the retrospective nature and limited sample size of this case series; however, written informed consent for the procedure and use of clinical data for academic purposes was obtained from the patients' legal guardians in all cases.

### 2.1 Indications and contraindications

Pulmonary artery (PA) stenting is primarily indicated in patients with congenital heart disease (CHD) who present with branch pulmonary artery stenosis or hypoplasia. Typical clinical scenarios include (1) Patients with right ventricular outflow physiology and cyanotic heart disease at the ventricular level complicated by branch PA stenosis, (2) Those who have undergone systemic-to-pulmonary shunt procedures or (3) Interventions such as Blalock–Taussig shunt, patent ductus arteriosus (PDA) stenting or right ventricular outflow tract (RVOT) stenting with subsequent branch PA stenosis or inadequate arterial growth with good distal runoff, and (4) Patients after bidirectional Glenn or Fontan procedures who develop branch PA stenosis. Additionally, patients with prior surgical reconstruction of the pulmonary arteries may require intervention if asymmetric growth of the pulmonary arteries is observed. Hemodynamic or imaging criteria further support intervention, including a significant pressure gradient across the PA (>20 mmHg) or a disproportionate flow distribution with a right-to-left pulmonary artery ratio <0.7 demonstrated by angiography, magnetic resonance imaging (MRI), or computed tomography (CT). Clinical parameters such as reduced systemic oxygen saturation or impaired pulmonary perfusion, particularly when a lung perfusion scan demonstrates unilateral perfusion of <30%, also constitute important indications.

Contraindications to PA stenting include both absolute and relative considerations. Absolute contraindications comprise active systemic infection or sepsis, severe renal dysfunction precluding the use of contrast agents, uncorrectable coagulopathy, severe contrast allergy, and markedly diminutive PA diameter (<2 mm) without adequate distal vessel arborization. Relative contraindications include very low body weight (<2 kg) and unstable clinical status despite inotropic support, which may limit procedural safety and efficacy.

### 2.2 Procedural technique

Written informed consent was obtained from all patients prior to the intervention. Cross-matched blood products were prepared in anticipation of possible transfusion requirements. All procedures were performed under general anesthesia with endotracheal intubation. Baseline hemodynamic parameters and oxygen saturation were recorded at room temperature, and detailed measurements were subsequently obtained via right and left heart catheterization. Strict aseptic preparation was carried out at the right and left inguinal regions as well as the right internal jugular site. Local anesthesia with 2% lidocaine was administered at the puncture site.

Vascular access was obtained through the most feasible route, depending on the patient's surgical history (e.g., post-BCPS), and could involve either arterial or venous entry. Access was achieved using the Seldinger technique or surgical cutdown, followed by sheath placement. Intravenous heparin was administered at a dose of 100 IU/kg. A Judkins Right guiding catheter was advanced to the main pulmonary artery (MPA), and selective pulmonary angiography was performed in frontal and lateral projections to delineate the site of obstruction and to measure proximal and distal vessel diameters.

Stent selection was based on patient body weight according to the Kirklin table. In small infants (3–6 months of age or <3.5 kg), coronary drug-eluting stents were preferred, while in older children and adults, vascular stents were utilized. Stent diameter was determined according to half-size Kirklin standards; for example, a 3-kg infant would typically receive a 4-mm stent, whereas patients weighing more than 18 kg would generally receive a 10-mm stent. A guidewire appropriate to the stent size was advanced into the distal pulmonary artery across the stenotic segment, and the guiding catheter was positioned as distally as possible. An exchange wire was then introduced, and the guiding catheter was withdrawn to allow advancement of the stent. The stent was deployed across the stenotic lesion, with predilation using a coronary or vascular balloon considered in cases of multilevel stenosis. The stent was expanded fully with the balloon catheter.

Following deployment, intravenous furosemide (1–1.5 mg/kg) and continuous milrinone infusion (0.375 mg/kg/min) were administered. Repeat hemodynamic measurements were obtained to assess the reduction in pressure gradient. A pullback maneuver was performed, and no significant pressure differences were observed in any patient; however, these measurements were not formally documented in the procedural reports. Post-stenting pulmonary angiography was performed to evaluate stent position and vessel anatomy, and echocardiography was used for further confirmation. The procedure was deemed successful if the stent was well positioned, oxygen saturation improved, and there was no significant pressure differences. Post-procedure, the patient was administered aspirin 5 mg/kg/day or clopidogrel 0.2 mg/kg/day.

At the conclusion of the intervention, repeat measurements of systemic hemodynamics, oxygen saturation, and hemoglobin concentration were obtained, with a target hemoglobin of 14 g/dL; transfusion was administered if necessary. All patients were monitored in the intensive care unit (ICU) for 24 h post-procedure and were typically discharged within one week if hemodynamic stability was maintained.

## 3 Results

**Figure 1:**
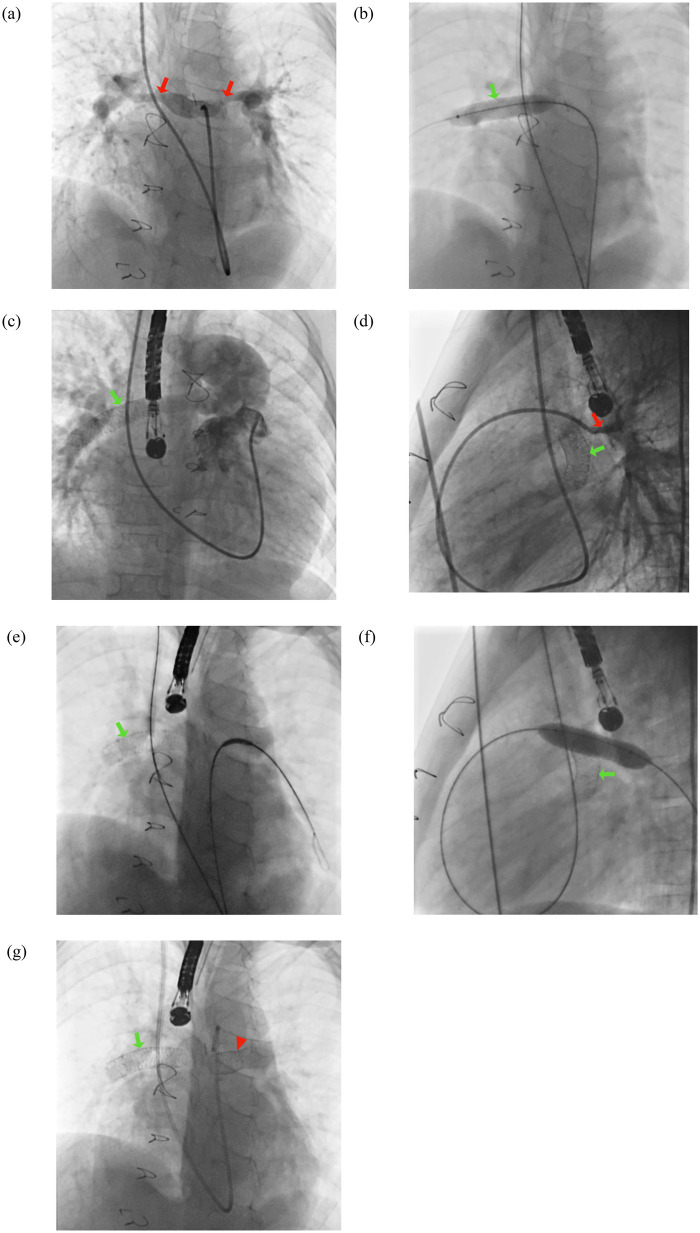
Bilateral pulmonary artery (PA) stenting procedure. **(a)** Angiographic visualization demonstrating bilateral PA stenosis. **(b)** Deployment of an Omnilink Elite (Abbott) 10.0 × 39 mm stent in the right pulmonary artery (RPA). **(c)** Post-stenting angiogram showing satisfactory flow in the RPA. **(d)** Angiogram of the left pulmonary artery (LPA) revealing proximal stenosis. **(e)** Positioning of an Omnilink Elite (Abbott) 10.0 × 29 mm stent in the LPA, followed by **(f)** balloon inflation. **(g)** Final angiographic result showing both stents in optimal position. The red arrow indicates the site of stenosis, the green arrow denotes the RPA stent, and the red arrowhead marks the LPA stent.

**Figure 2:**
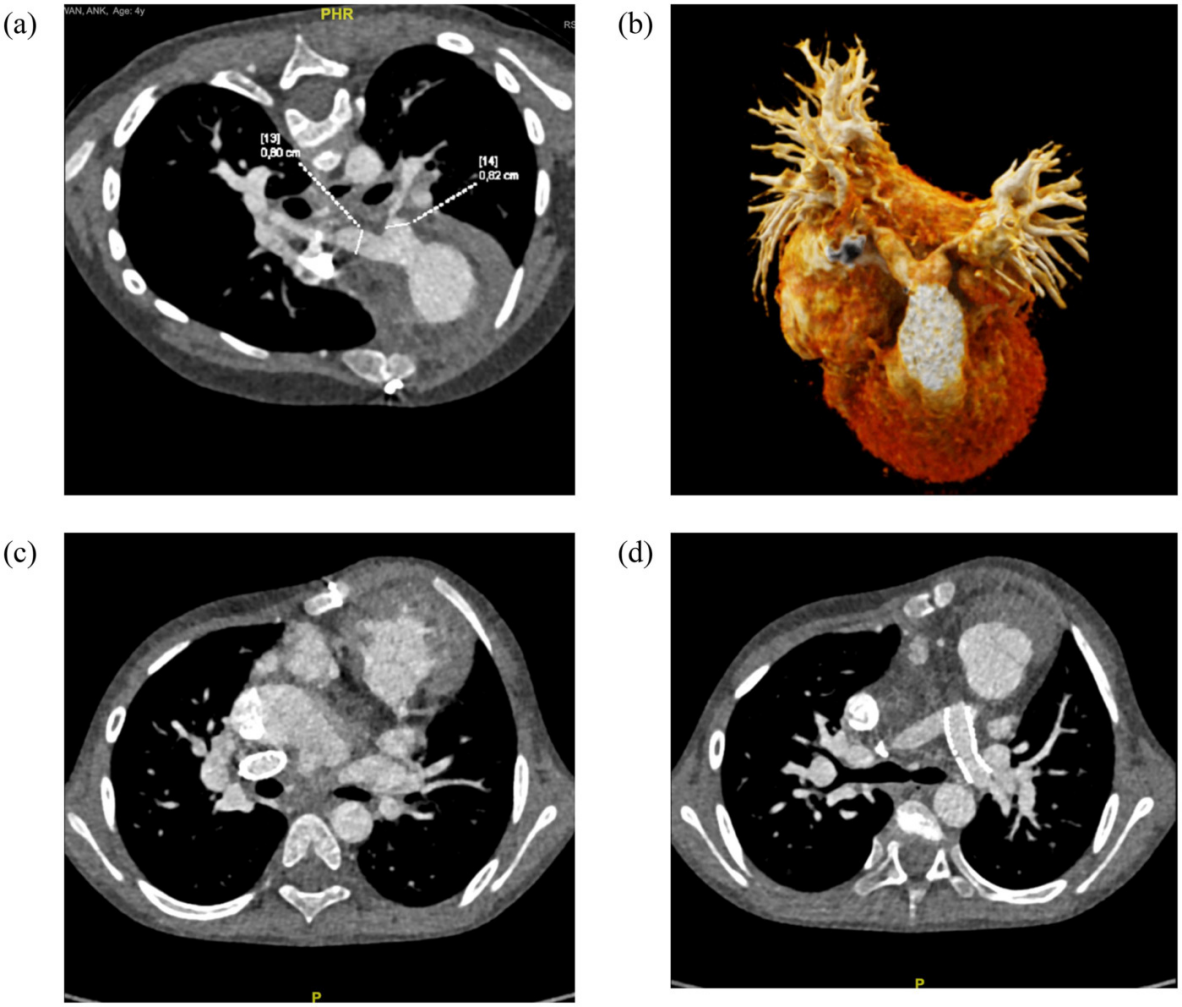
Follow-up CT imaging of a patient who underwent bilateral pulmonary artery (PA) stenting. **(a)** Baseline measurements of the left (LPA) and right pulmonary artery (RPA) diameters. **(b)** Three-dimensional reconstruction demonstrating bilateral PA stenosis prior to intervention. **(c)** Post-stenting appearance of the RPA. **(d)** Post-stenting appearance of the LPA.

A total of 18 patients underwent pulmonary artery (PA) stenting during the study period, comprising 10 left pulmonary artery (LPA), seven right pulmonary artery (RPA) interventions, and one patient had bilateral PA stenting (see Figures 1, 2). The median age at the time of procedure was 5.4 years (range: 3 months–17 years), and the cohort was predominantly female (77.78%). The most common underlying diagnoses were tetralogy of Fallot (ToF) with or without prior repair, double outlet right ventricle (DORV), and single ventricle physiology after bidirectional cavopulmonary shunt (BCPS) or Fontan circulation (**see** Table 1).

**Table 1:** Baseline characteristics of patient who had pulmonary artery stenting.

No	Gender	Age	Diagnosis	Body weight (kg)	Location of the pulmonary artery stenosis	Approach	Stent type and size	Sheath size (F)	Saturation before (%)	Saturation during follow-up (%)	Successful stent deployment	Fluoroscopy time (mins)	DAP (uGym2)	Complication	Recent follow-up
LPA stent															
1	F	17 years	d-TGA, VSD, PS s/p R-BCPS	47	Mid-LPA	Antegrade transjugular	Dynamic vascular stent 10 × 38 mm	5	89	94	Y	13	1,871.5	None	Patient's condition was good
2	F	2 years 2 months	ToF s/p L-BT shunt	14.5	Severe stenosis at proximal LPA	Antegrade transfemoral	Abbott Vascular Omnilink Elite 10.0 mm × 19 mm	5	70	99	Y	30.3	942.86	None	Patient has undergone total repair one-year post-procedure
3	F	1 year 3 months	Residual stenosis s/p ToF repair	14	Stenosis in proximal LPA and RPA with MPA dilatation	Antegrade transfemoral	Omnilink Elite (Abbott) 7.0 × 29 mm	7	99	99	Y	24.1	571.07	Stent dislodgement to main pulmonary artery	Patient has undergone stent evacuation and pulmonary artery repair. Patient's condition was good
4	F	1 year 9 months	DORV, CAVSD, PS, PDA, hypoplastic LV s/p PDA stenting, s/p BCPS, repair of the bifurcation and proximal RPA with a pericardial patch, partial stent removal, division of the PDA, division of the MPA and azygos vein.	8	Severe stenosis at proximal LPA	Antegrade transjugular	Dynamic vascular stent (Biotronik AG) 10 × 38 mm	8	54	N/A	Success on deployment, but patient had cardiac arrest episode on the cathlab	N/A	N/A	Cardiac arrest	Death
5	F	5 years 1 month	DORV, MA, hypoplastic LV, muscular VSD, common atrium, valvar PS, PDA, bilateral SVC. s/p bilateral BCPS, division and suturing of the MPA, division and suturing of the PDA, and ligation of the left azygos vein	16	Stenosis at proximal LPA	Antegrade transjugular	Dynamic vascular stent (Biotronik AG) 10 × 38 mm	8	64	77	Y	5.4	N/A	None	Patient has undergone fenestrated Fontan procedure
6	F	3 months	Situs invs., ventricular inversion, double inlet left ventricle (DILV), pulmonary atresia with ventricular septal defect (PA-VSD), vertical PDA, secundum ASD, bilateral SVC, hypoplastic right ventricle, severe tricuspid regurgitation, mild mitral regurgitation, and mild-to-moderate aortic regurgitation	6.2	Stenosis in bifurcation of MPA	Retrograde transcarotid	Promus Premiere 4.0 mm × 20 mm	5	69	78	Y	4.5	174.41	Sepsis	Patient died post BCPS in the intensive care unit
7	F	5 years 8 months	Post bilateral BCPS, ligation of the azygos and hemiazygos veins, ligation of the PDA, division of the MPA, and atrial septectomy; s/p IAS stenting; s/p PA banding and PDA ligation in mitral atresia, DORV, inlet-to-outlet VSD, and restrictive PFO	18.7	Stenosis at proximal LPA	Antegrade transjugular	Omnilink Elite (Abbott) 10.0 × 29 mm	7	82	98	Y	13.7	157.67	None	Patient has undergone fenestrated Fontan procedure
8	M	12 years 11 months	Mesocardia, situs solitus, ventricular inversion, tricuspid atresia, pulmonary atresia, inlet VSD, secundum ASD, PDA, collateral vessels; status post BCPS, pulmonary hypertension, and status post embolization of MAPCAs	59	Stenosis at mid-LPA	Antegrade transjugular	Omnilink Elite (Abbott) 10.0 × 29 mm	8	90	98	Y	6.5	1,536.01	None	Patient has undergone fenestrated Fontan procedure
9	F	4 years 9 months	Pulmonary atresia with VSD (PA-VSD), PDA, bilateral SVC, PFO; status post PDA stenting; status post right modified Blalock–Taussig–Thomas (BTT) shunt, left pulmonary artery repair, division of PDA, and stent removal following left thoracotomy; left BTT shunt, extraction of residual PDA stent, and division of remaining PDA	14.2	Stump in LPA	Retrograde transfemoral	Onyx Trucor 4.0 mm × 30 mm	5	80	94	Y	72.3	1,731.32	None	Patient's condition was good
10	F	6 years 4 months	Tricuspid atresia, pulmonary atresia with intact ventricular septum (IVS), PFO, hypoplastic right ventricle, PDA; status post right BCPS, atrial septectomy, repair of the LPA–RPA with a pericardial patch, division of the PDA, and division of the azygos vein for total occlusion at the proximal LPA near the PDA bifurcation	15.2	Stenosis in proximal LPA	Antegrade transjugular	Omnilink Elite (Abbott) 8.0 × 29 mm	5	73	92	Y	35.2	705.8	None	Patient's condition was good
Bilateral PA stent															
11	F	6 years 2 months	Situs solitus, ventricular inversion, pulmonary atresia, large inlet VSD, large secundum ASD, hypoplastic mitral valve, hypoplastic left ventricle, aorta arising from the right ventricle, right aortic arch, distal hypoplasia of the right and left pulmonary arteries; status post central shunt	18	Stenosis in proximal LPA and distal RPA	Antegrade transjugular	Omnilink Elite (Abbott) 10.0 × 29 mm (LPA) and Omnilink Elite (Abbott) 10.0 × 39 mm (RPA)	7	89	96	Y	16.24	1,325.94	None	Patient's condition was good
RPA stent															
12	F	4 years	Stenosis at the origin of the right pulmonary artery, residual VSD with left-to-right shunt in a patient status post TOF repair with transannular patch (TAP), repair of the left and right pulmonary arteries with pericardial patch, residual ASD measuring 5 mm, takedown of left and right BT shunts, and status post left diaphragmatic plication	18	Stenosis in pulmonary artery bifurcation	Antegrade transfemoral	Omnilink Elite 10 mm × 29 mm	8	99	99	Y	6.7	365.96	Diaphragma paralysis, acute kidney injury, septic shock	Death
13	F	12 years 4 months	Dextrocardia, situs invs., pulmonary atresia, complete atrioventricular septal defect (CAVSD), aorta arising from the right ventricle, hypoplastic left ventricle; status post bilateral BCPS, MAPCA embolization, and proximal right pulmonary artery stenosis.	40	Stenosis in proximal LPA and RPA	Antegrade transjugular	Dynamic Vascular Stent 10 × 38 mm	5	78	80	Y	6.42	434.66	Inadequate stent inflation due to external compression	Patient easily gets tired when walking long distances.
14	M	2 years 8 months	Post TOF repair with residual VSD and ASD/PFO due to hypoplastic right pulmonary artery (long segment stenosis from the bifurcation to the hilum)	11.5	Hypoplastic right pulmonary artery (long segment stenosis from the bifurcation to the hilum)	Antegrade transfemoral	Omnilink Elite (Abbott) 10 mm × 29 mm × 135 mm	8	77	97	Y	15.3	702.34	None	Patient's condition was good
15	M	5 years 5 months	Mesocardia, situs ambiguus, right atrial isomerism, double outlet right ventricle (DORV), muscular inlet VSD, severe valvar and subvalvar pulmonary stenosis, malposed great arteries; status post failed RVOT stenting, status post right BT shunt, and status post left BCPS.	15.3	Severe stenosis in proximal RPA	Antegrade transjugular	Omnilink Elite 10 × 29 mm, followed by placement of another Vascular Omnilink Elite 10 × 29 mm stent in an overlapping position	8	92	90	Y	10.55	287.79	None	Patient has undergone fenestrated Fontan procedure
16	F	14 years 11 months	Severe stenosis at the proximal right pulmonary artery; status post TOF repair with transannular patch (TAP), VSD closure, infundibular and valvular pulmonary stenosis resection, and main pulmonary artery repair with TAP; residual VSD measuring 3 mm, PFO creation; status post balloon dilatation of the right and left pulmonary arteries	42.3	Stenosis in proximal RPA	Antegrade transfemoral	Omnilink Elite 10.0 × 39 mm	8	99	99	Y	5.39	466.15	None	Patient's condition was good
17	M	9 months 3 months	Situs ambiguus, right atrial isomerism, double outlet right ventricle (DORV), large inlet VSD, critical pulmonary stenosis, right aortic arch; status post left BCPS and Fontan procedure with fenestration performed.	35	Stenosis in proximal RPA	Antegrade transfemoral	Omnilink Elite 10.0 × 29 mm	7	64	80	Y	7.45	957.1	None	Patient's condition was good
18	F	10 years 8 months	Tetralogy of Fallot with right aortic arch and bilateral superior vena cavae; status post TOF repair with reconstruction of the proximal right pulmonary artery and ASD creation	22.2	Stenosis at the proximal to mid right pulmonary artery.	Antegrade transfemoral	Omnilink Elite 10.0 × 19 mm	8	96	96	Y	24.56	602.75	None	Patient's condition was good

### 3.1 Procedural characteristics

The majority of procedures were conducted using an antegrade approach, either through the transjugular (*n* = 9) or transfemoral (*n* = 7) route. Retrograde access, via the transcarotid or transfemoral approach, was employed in two cases. The most commonly utilized stents included the Omnilink Elite (Abbott Vascular; *n* = 14) and Dynamic Vascular Stent (Biotronik AG; *n* = 4) with sheath sizes between 5F and 8F.

Two cases required the use of two stents. The first involved bilateral pulmonary artery (PA) stenting in a six-year-old girl (Patient 11), in whom Omnilink Elite stents (Abbott) were deployed, 10.0 × 29 mm in the left pulmonary artery (LPA) and 10.0 × 39 mm in the right pulmonary artery (RPA). The second case was a five-year-old boy (Patient 15) with a history of left bidirectional cavopulmonary shunt, who underwent double stenting using Omnilink Elite (Abbott) 10.0 × 29 mm stents placed in an overlapping configuration. Both patients demonstrated favorable outcomes without procedure-related complications.

### 3.2 Hemodynamic and clinical outcomes

Successful procedural stent deployment was achieved in all cases. The mean pre-procedural oxygen saturation was 78.1% ± 12.5, which improved to 91.6% ± 7.3 during follow-up. Fluoroscopy time ranged from 4.5 to 72.3 min, while the dose–area product (DAP) varied from 153.7 to 1871.5 μGy·m², reflecting differences in anatomical complexity and vascular access routes.

A total of five complications, mostly unrelated to stenting procedure were recorded. Three patients experienced major adverse events resulting in death. Patient 4 suffered cardiac arrest after stent implantation; notably, this patient had a history of recurrent cardiac arrest episodes during hospitalization, suggesting that the event was primarily attributable to the patient's deteriorating clinical condition rather than the procedure itself. Patient 6 developed septic shock and multiorgan failure after bidirectional cavopulmonary shunt (BCPS). Despite intensive management, the patient's condition worsened, with progressive bradycardia (60–88 beats per minute), severe desaturation (20%–33%), and worsening acidosis on venous blood gas analysis, leading to cardiac arrest and death in the intensive care unit. Patient 12 developed diaphragmatic paralysis, acute kidney injury, and septic shock leading to death, which were considered unrelated to the intervention, but rather associated with the patient's underlying condition as a post–Tetralogy of Fallot (ToF) repair case.

Patient 3 experienced stent dislodgement that required surgical retrieval. In Patient 13, suboptimal stent expansion occurred due to increased arterial stiffness. The patient declined additional interventional management and currently reports exertional fatigue during extended ambulation. No occurrences of vascular rupture or pericardial tamponade were identified in this cohort.

### 3.3 Follow-up

During a median follow-up of 12 months, most surviving patients demonstrated good clinical status and sustained oxygen saturation improvement. Several patients subsequently underwent definitive surgical repair or Fontan completion following successful branch PA stenting.

## 4 Discussion

### 4.1 Importance of pulmonary artery stenting

Pulmonary artery (PA) stenting has become an indispensable component in the management of congenital heart disease (CHD), particularly in patients who develop branch pulmonary artery stenosis following palliative or corrective procedures such as the Blalock–Taussig (BT) shunt, right ventricular outflow tract (RVOT) stent, patent ductus arteriosus (PDA) stent, bidirectional cavopulmonary shunt (BCPS), or Fontan (2, 3, 5). These lesions sometimes cause unequal pulmonary blood flow, persistent cyanosis, and increased ventricular workload, which, if left uncorrected, can impair the success of subsequent surgical stages such as Fontan completion or definitive repair (7, 8). In this clinical context, PA stenting serves as both a palliative and preparatory intervention that improves vessel patency, enhances systemic oxygenation, and optimizes pulmonary hemodynamics.

Compared with balloon angioplasty alone, PA stenting provides more durable outcomes because the stent exerts a radial force that prevents elastic recoil and late restenosis (1). Balloon angioplasty, while useful for smaller or distal branches, often results in recurrent obstruction within one year in more than 10% of cases, as previously reported in the literature (3). The mechanical stability provided by the stent ensures long-term patency and better flow distribution in the pulmonary arteries (3, 4). For this reason, stenting is particularly valuable in patients with complex or postoperative anatomies where repeat surgery poses considerable risk (3, 13–16). The high procedural success rate in this series supports the growing role of PA stenting as a safe and effective therapy that bridges the gap between palliation and definitive correction in complex congenital heart disease.

### 4.2 Clinical outcomes and complications

In this study, PA stenting achieved a procedural success rate of 88.9%, accompanied by a marked improvement in systemic oxygen saturation during follow-up. This demonstrates that the procedure effectively restores pulmonary blood flow and contributes to overall clinical stability (1, 3). Most patients subsequently progressed to their next surgical stage, including Fontan completion or total repair, confirming the procedure's importance in staged surgical management (2, 7).

Despite the high success rate, several complications occurred, reflecting both the anatomical complexity and physiological fragility of this patient population. Cardiac arrest occurred in a patient with single ventricle physiology and extensive prior surgeries. In such cases, abrupt hemodynamic changes during the procedure, such as sudden restoration of pulmonary blood flow or rapid contrast injection, can precipitate acute ventricular dysfunction or arrhythmia (17, 18). Preventive measures include gradual reperfusion of the pulmonary circuit, careful preload control, and close hemodynamic monitoring during anesthesia (19, 20). Another mortality was attributed to sepsis in a critically ill infant with prolonged postoperative care. This emphasizes that the risk of infection is amplified in cyanotic patients due to chronic hypoxia, malnutrition, and prolonged indwelling catheters. Strict aseptic technique, prophylactic antibiotic use, and early recognition of systemic infection are therefore vital components of peri-procedural management (21).

Stent dislodgement occurred in one patient, necessitating surgical retrieval. This complication is commonly associated with inadequate anchoring or inaccurate assessment of the landing zone, particularly in regions with high-pressure gradients or irregular vessel anatomy (1, 3, 10, 12). Thorough pre-procedural angiographic evaluation, careful selection of slightly oversized stents, and controlled balloon inflation are essential to ensure secure deployment and prevent migration. Inadequate stent expansion may also occur in over-compliant or irregular pulmonary arteries, leading to incomplete apposition and residual stenosis. In such cases, excessive vessel compliance allows outward wall deformation during balloon inflation, while eccentric or tortuous anatomy creates uneven radial forces that impair uniform expansion. Technical factors such as insufficient predilation, inappropriate balloon selection, or suboptimal coaxial positioning may further contribute to suboptimal outcomes. Management involves reassessment of stent position and vessel morphology using orthogonal angiography or intravascular imaging, followed by high-pressure post-dilation with non-compliant balloons of appropriate size. If a residual waist persists, sequential balloon upsizing, deployment of a higher-radial-force stent using a stent-in-stent technique, or selective flaring of stent ends may be required to achieve optimal luminal expansion (1, 22–24).

### 4.3 Stent selection and landing zone considerations

Stent selection and landing zone identification are key factors that determine procedural success, long-term patency, and the need for future reintervention. In this study, the most frequently used devices were the Omnilink Elite (Abbott Vascular) and Dynamic Vascular (Biotronik AG) stents, chosen for their compatibility with smaller delivery systems (5–8F) and ease of deployment in infants and small children. Although premounted stents provide excellent deliverability, their limited capacity for post-dilation to adult diameters restricts their long-term adaptability. Nevertheless, they remain the preferred option for small or hemodynamically unstable patients, where the immediate priority is to restore blood flow safely (25).

For older children and adolescents, non-premounted expandable stents such as the Cheatham-Platinum (CP) stent are advantageous because they can be redilated to accommodate somatic growth up to 24 mm in diameter. However, their need for larger delivery sheaths (10–12F) may preclude their use in younger patients. In this context, hybrid techniques, combining surgical exposure with catheter-based stent delivery, can be particularly useful when large stents must be implanted in small patients or in those with limited vascular access (26).

Landing zone selection is equally critical to procedural success. Proximal PA lesions are subjected to high pressure and flow, increasing the risk of stent migration or incomplete expansion, while distal or lobar lesions carry the potential for reperfusion injury due to abrupt restoration of flow. Accurate angiographic mapping of proximal and distal vessel diameters, adequate guidewire support, and an understanding of prior surgical patches or suture lines are essential for safe and effective deployment. Proper landing zone selection not only ensures immediate procedural success but also facilitates future interventions such as redilation or re-stenting if required (25, 26).

## 5 Limitations

This study has several limitations. First, it represents a retrospective, single-center experience with a relatively small sample size, which limits the generalizability of the findings. The heterogeneous nature of the patient population, including varying anatomical diagnoses, surgical backgrounds, and hemodynamic conditions, makes it difficult to perform subgroup comparisons or identify independent predictors of outcome.

The choice of stent type and procedural approach was based on operator preference and individual anatomy rather than standardized criteria, reflecting real-world clinical decision-making but introducing potential selection bias. Additionally, certain hemodynamic variables, such as distal pulmonary artery pressures or perfusion scan results were unavailable in all patients, preventing complete quantitative analysis of post-procedural improvement. Future multicenter, prospective studies with standardized imaging and long-term follow-up are needed to confirm the durability and safety of pulmonary artery stenting in this diverse patient population.

## 6 Conclusions

In this retrospective case series, pulmonary artery stenting was feasible in selected pediatric patients with branch pulmonary artery stenosis and complex congenital heart disease. The intervention was associated with satisfactory short-term procedural outcomes, including improvement in vessel patency and clinical status in most patients. However, given the limited sample size, retrospective design, and occurrence of mortality during follow-up, likely related to underlying disease complexity rather than the procedure itself, definitive conclusions regarding overall effectiveness cannot be drawn. Pulmonary artery stenting may serve as a useful adjunctive or bridging strategy following procedures such as BT shunt, RVOT stenting, or BCPS, potentially facilitating subsequent surgical stages. Procedure-related complications were observed and underscore the importance of careful patient selection, individualized stent choice, and meticulous procedural and post-procedural management. Larger, prospective studies with longer follow-up are required to better define the safety profile and long-term impact of this intervention.

## Data Availability

The original contributions presented in the study are included in the article/Supplementary Material, further inquiries can be directed to the corresponding author.
